# Interfaces between cellular responses to DNA damage and cancer immunotherapy

**DOI:** 10.1101/gad.348314.121

**Published:** 2021-05-01

**Authors:** Domenic Pilger, Leonard W. Seymour, Stephen P. Jackson

**Affiliations:** 1 Wellcome Trust/Cancer Research UK Gurdon Institute, Department of Biochemistry, University of Cambridge, Cambridge CB2 1QN, United Kingdom;; 2 Department of Oncology, University of Oxford, Oxford, Oxford OX3 7DQ, United Kingdom

**Keywords:** DNA damage response, DNA repair, immunotherapy, PARP inhibitors, PD-1, PD-L1, STING, cGAS

## Abstract

The DNA damage response (DDR) fulfils essential roles to preserve genome integrity. Targeting the DDR in tumors has had remarkable success over the last decade, exemplified by the licensing of PARP inhibitors for cancer therapy. Recent studies suggest that the application of DDR inhibitors impacts on cellular innate and adaptive immune responses, wherein key DNA repair factors have roles in limiting chronic inflammatory signaling. Antitumor immunity plays an emerging part in cancer therapy, and extensive efforts have led to the development of immune checkpoint inhibitors overcoming immune suppressive signals in tumors. Here, we review the current understanding of the molecular mechanisms underlying DNA damage-triggered immune responses, including cytosolic DNA sensing via the cGAS/STING pathway. We highlight the implications of DDR components for therapeutic outcomes of immune checkpoint inhibitors or their use as biomarkers. Finally, we discuss the rationale for novel combinations of DDR inhibitors with antagonists of immune checkpoints and current hindrances limiting their broader therapeutic applications.

## Abstract (precis)

In this review, Pilger et al. summarize the current understanding of the molecular mechanisms underlying DNA damage-triggered immune responses, including cytosolic DNA sensing via the cGAS/STING pathway. They also highlight the implications of DDR components for therapeutic outcomes of immune checkpoint inhibitors or their use as biomarkers.

## Targeting DNA damage response factors for cancer therapy

**Figure 1.**
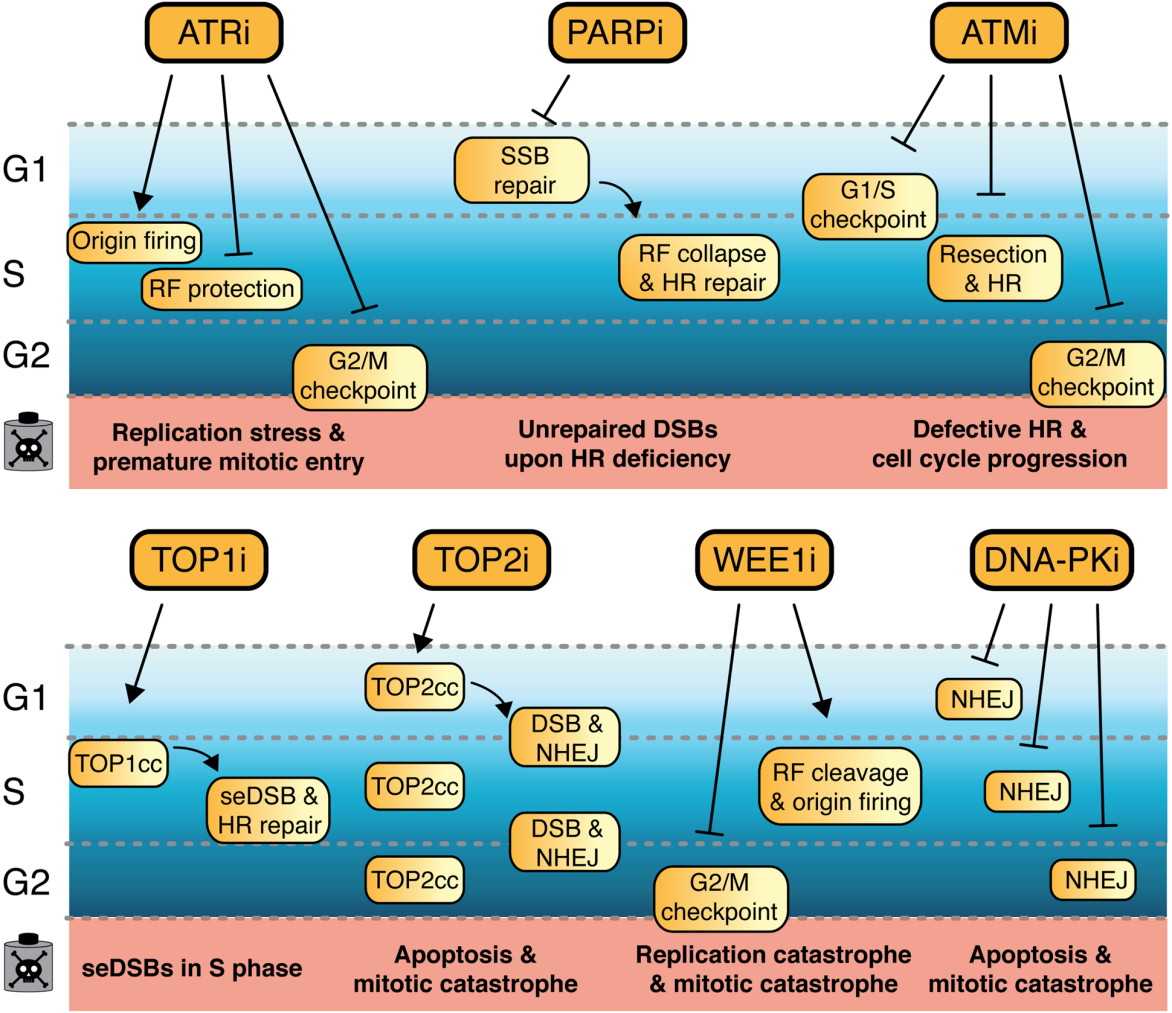
DDR inhibitors and their impacts on DSB repair and cell cycle progression. In mammalian cells, four main DSB pathways exist, which operate dependent on the stage of the cell cycle: nonhomologous end-joining (NHEJ), alternative end-joining (alt-EJ), homologous recombination (HR), and single-strand annealing (SSA) (Scully et al. 2019). TOP2i induced DSBs are predominantly repaired via NHEJ, which while being described as “error-prone,” ensures effective repair of broken DNA ends particularly during G0 and in the G1 phase of the cell cycle. DNA-PK is crucial for effective NHEJ, and DNA-PK inhibitors impair DSB repair via NHEJ. TOP1 inhibitors generate single-ended DSBs (seDSBs) in S phase, which require HR for accurate repair. HR is a form of DNA recombination where DNA homology and synthesis can accurately regenerate the sequence surrounding the DSB, facilitated by a sister chromatid as template and therefore restricted to S or G2 (Karanam et al. 2012). DNA damage arising from PARP inhibition also requires HR, since spontaneously occurring SSBs are converted into seDSBs during DNA replication upon PARP inhibitors (PARPis). In addition, PARPi cause mitotic catastrophe and induce DNA replication stress through altering DNA replication fork speed. A fundamental step during HR-mediated repair is DNA end resection, generating ssDNA overhangs that are rapidly covered by RPA and consequently by RAD51. ATR is activated by ssDNA as a result of DNA end resection or DNA polymerase uncoupling from helicase activity during DNA replication. Consequently, ATR inhibitors primarily have impacts during S and G2 phases of the cell cycle. Additionally, ATRi overrides the G2/M cell cycle checkpoint, therefore causing premature entry into mitosis. Importantly, ATM inhibition affects efficient HR, alongside its impact on G1/S and G2/M cell cycle checkpoints in response to DNA damage. Analogous to ATRi and ATMi, WEE1 inhibitors affect the G2/M cell cycle checkpoint. Moreover, WEE1i cause replication stress through dysregulated origin firing and cleavage of DNA replication forks, resulting in DSBs.

The genome of every cell is constantly exposed to endogenously-arising and exogenous sources of DNA damage. To ensure genome stability and faithful replication and transmission of the genetic material, various DNA repair pathways have evolved to allow cellular and organism survival. This complex network of DNA damage sensor, mediator, and effector proteins is known as the DNA damage response (DDR) (Ciccia and Elledge 2010). The DDR exhibits tight spatiotemporal control, ensuring the precise and proper actions and coordination of repair enzymes in a DNA lesion-specific manner. Unscheduled or uncontrolled activity of DNA processing and repair factors can themselves generate DNA damage, thereby posing threats to genome integrity and cell survival. Consequently, dysregulation and mutations in such DDR factors and their regulators have implications for human health and disease (Jackson and Bartek 2009). Moreover, in recent years, DDR components have become accepted as attractive therapeutic targets for cancer therapy, with numerous small molecule inhibitors targeting DNA repair enzymes now being explored clinically and with inhibitors of the DNA-repair enzyme PARP being approved for treating various tumor types (Piliè et al. 2019). Due to the frequent loss or deregulation of DDR mechanisms and high levels of DNA replication stress caused by oncogene activation, cancer cells often display elevated levels of endogenous DNA lesions while simultaneously relying on certain DNA repair pathways for survival (Hanahan and Weinberg 2011). This enhanced dependence on certain DDR components—sometimes via the concept of synthetic lethality, where loss of one cellular pathway leads to reliance on an alternative pathway—came into the spotlight with the development of poly-(ADP-ribose) polymerase (PARP) inhibitors as precision medicines for certain cancers harboring defects in the DDR mechanism of homologous recombination (Lord and Ashworth 2017). PARP enzymes (mainly PARP1 and PARP2) fulfill various functions during DNA repair, but in particular, PARP1 is important for the effective repair of DNA single-strand breaks (SSBs). Notably, alongside inhibition of SSB repair, PARP inhibitors compete with the cofactor NAD^+^, thereby preventing poly-ADP-ribosylation (PARylation) of PARP1 itself and various other proteins. Because auto-PARylation promotes the release of PARP1 from DNA, PARP inhibitors result in PARP1 becoming “trapped” on DNA. Importantly, much of the cytotoxicity of PARP inhibitors has been attributed to when DNA replication forks encounter these trapped PARP–SSB complexes, leading to replication fork collapse and generation of DNA double-strand breaks (DSBs), specifically single-ended DSBs (a DSB end with no associated DNA end to be ligated to). The potential to exploit this scenario became apparent when two independent studies reported dramatic cytotoxicity of PARP inhibitors in the context of deficiencies in the tumor suppressor genes *BRCA1* and *BRCA2* (breast cancer susceptibility genes 1 and 2) (Bryant et al. 2005; Farmer et al. 2005). BRCA1 and BRCA2 have crucial functions during homologous recombination (HR)-mediated repair of DSBs (Fig. 1). While PARP inhibition is dispensable for cellular survival in HR-proficient cells, it becomes extremely toxic in the context of BRCA1/2 deficiency, highlighting the key role of HR in repairing PARP inhibitor-induced damage (Lord and Ashworth 2016). So far, four different PARP inhibitors (olaparib, niraparib, rucaparib, and talazoparib) have been approved by the FDA for use as single-agent chemotherapeutics (Table 1).

**Table 1.**
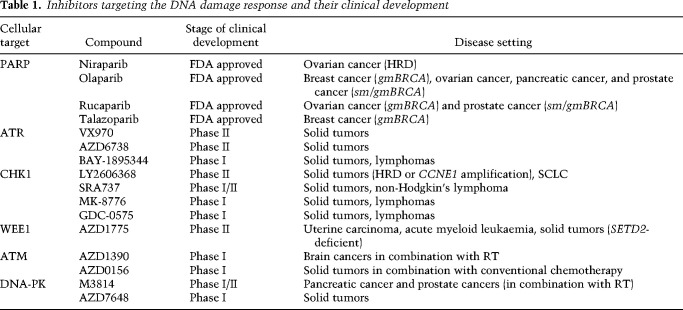
Inhibitors targeting the DNA damage response and their clinical development

The clinical successes of PARP inhibitors have nurtured efforts to target other components of the DNA repair network for therapeutic applications. Thus, the three apical PI3K-like kinases ATM, ATR, and DNA-PK, in addition to the S-phase checkpoint kinase CHK1 and G2 checkpoint kinase WEE1, have emerged as promising targets for small molecule inhibitors, with several of these compounds currently undergoing clinical development (Blackford and Jackson 2017; Forment and O'Connor 2018). The justification for targeting ATR in cancer cells centers around ATR's functions in protecting stalled DNA replication forks, regulating replication origin firing, and controlling the transition of cells from G2 phase into mitosis by enforcing the G2/M cell cycle checkpoint (Saldivar et al. 2017). Due to their proliferative nature and being subject to oncogenic forces that either lead to heightened levels of reactive oxygen species or deregulated S-phase entry and/or progression, cancer cells are particularly vulnerable to ATR inhibition, experiencing extensive DNA and chromosomal damage. Furthermore, it has been found that ATR inhibitors are selectively toxic to cancer cells harboring mutations in the *ATM* tumor suppressor, in part because ATM and ATR share some overlapping targets (Kantidze et al. 2018). Additionally, ATM loss impairs DSB repair by HR and also weakens the G1/S cell cycle checkpoint, thereby generating more replicative stress (Lecona and Fernandez-Capetillo 2018). Several ATR inhibitors are currently undergoing clinical development, with their application mainly aiming to exploit high levels of replication stress in tumors. Since CHK1 is the effector kinase of ATR, several cellular functions are shared between these kinases, and the rationales behind exploring CHK1 inhibitors for cancer therapy are hence similar to those for ATR inhibitors (Table 1; Forment and O'Connor 2018).

WEE1 is involved in responses to DNA damage, whereby it enforces a cell cycle arrest/checkpoint at the transition from S phase into M phase in response to DNA damage or replication stress. WEE1 phosphorylates and inhibits cyclin-dependent kinases (CDKs) CDK1 and CDK2**,** thus counteracting cell cycle progression and unscheduled replication-origin firing (Elbæk et al. 2020). One of the concepts for the clinical application of WEE1 inhibitors is that WEE1 inhibition should potentiate the effects of certain other DNA damage-inducing chemotherapeutics, since cells undergoing WEE1 inhibition would enter mitosis with DNA lesions or underreplicated DNA, thereby causing mitotic catastrophe and ensuing cell death. Indeed, the efficacy of WEE1 inhibition in combination with other DNA-damaging agents such as platinum drugs or irradiation has been observed in preclinical model systems (Hirai et al. 2009; PosthumaDeBoer et al. 2011).

DNA-PK and ATM are key protein kinases that function in the DDR to promote DNA nonhomologous end-joining (NHEJ) and HR, respectively, with ATM also playing major roles in regulating signaling cascades in response to irradiation and other DNA-damaging agents (Blackford and Jackson 2017). Because their absence or inhibition sensitizes cells to irradiation, PARP inhibitors, and various DNA-damaging chemotherapeutic agents, there is the potential for using ATM or DNA-PK inhibition to enhance antitumor efficacies of radiotherapy and certain chemotherapies, although their potential to also accentuate toxic side effects in patients will have to be carefully managed in such contexts (Table 1).

In contrast to DDR enzyme inhibitors, other classes of drugs possess the potential to generate DNA damage and therefore are classed as genotoxic agents. Among them, topoisomerase inhibitors are well-established chemotherapeutics for a variety of cancers. The topoisomerase enzymes TOP1 and TOP2 generate transient DNA breaks in order to resolve topological stresses during DNA replication and transcription, using transesterification reactions to break the DNA phosphodiester backbone while at the same time forming a transient covalent bond between the enzyme's catalytic tyrosine (Tyr) and the DNA, known as the topoisomerase cleavage complex (TOPcc). Exploiting the potential of topoisomerases to induce DNA damage, especially in highly replicating and transcribing cells, underpinned the development of several classes of TOP1 and TOP2 inhibitors. Among others, molecules that reversibly stabilize TOPccs to block the religation reaction have had dramatic impacts as chemotherapeutic agents. Since these molecules act as interfacial inhibitors (functioning via stabilizing a reaction intermediate), DNA damage arises from TOP1 or TOP2 that has become trapped in the state where the DNA backbone is already cut and the enzyme cross-linked to DNA, but the reverse reaction is abrogated (Hsiang et al. 1985; Covey et al. 1989; Pommier 2009). In the context of TOP1, trapped TOP1cc becomes particularly toxic in replicating cells when DNA replication forks encounter these lesions, leading to replication fork arrest, fork collapse, and eventually DNA DSB formation (Hsiang et al. 1989; Strumberg et al. 2000; Furuta et al. 2003). Although camptothecin was among the first TOP1 inhibitors to be identified, its clinical application was not pursued further, and the camptothecin derivatives topotecan and irinotecan instead gained regulatory approval (Table 1). Clinically approved TOP2 inhibitors, such as etoposide and doxorubicin (Table 1), function as interfacial inhibitors by trapping the TOP2ccs on DNA and preventing religation of the DNA backbone, analogous to the actions of camptothecin derivatives on TOP1 (Wu et al. 2011, 2013a). However, due to TOP2's dimeric mode of action, key aspects of TOP2 inhibitors’ mechanism are their ability to directly induce DSBs and consequently yield such toxic lesions in all stages of the cell cycle (Holm et al. 1989).

Platinum-based chemotherapeutics represent another class of genotoxic agents, which have found broad application in cancer therapy. Initially, cisplatin was FDA approved for the treatment of testicular and ovarian cancers in 1978, whereas nowadays cisplatin and its derivates carboplatin and oxaliplatin are established chemotherapeutic agents for various tumor types (Kelland 2007). Platinum-based agents create DNA monoadducts and DNA cross-links, which impair cellular processes such as DNA replication and transcription and require specific DNA repair pathways for their resolution. As a consequence, platinum agents are strong inducers of cell cycle arrest and apoptosis (Rottenberg et al. 2021). Mechanistically, cisplatin is activated intracellularly through the aquation of the chloride group(s) and subsequently chemically cross-link DNA molecules, preferably at N7 or O6 in purines. Notably, platinum agents can cross-link proteins to DNA, although the occurrence of this type of lesion is much rarer compared with DNA intra-strand and inter-strand cross-links (Siddik 2003). Alongside topoisomerase inhibitors and platinum drugs, radiation therapy can be classified as a genotoxic treatment and represents the most frequently applied cancer therapy aside from surgery. The idea of radiation therapy is based on the locally targeted induction of DNA damage in order to deprive cancer cells of their proliferative potential by forcing them into senescence, apoptosis, or other forms of cell death (Bernier et al. 2004). Since radiation therapy induces a variety of DNA lesions, including DSBs, SSBs, base damage, and cross-links, the application of various DDR enzyme inhibitors can further potentiate the cytotoxic effects of radiation therapy (Moding et al. 2013).

## Sensing damaged DNA by components of the innate immune system

Alongside the immediate cytotoxic effects and persistent DNA damage caused by certain DDR inhibitors (DDRis), induction of innate immune responses has frequently been observed in such settings. Unrepaired DNA lesions and/or impaired chromosome segregation during mitosis contribute to the formation of endogenous cytosolic DNA, often in the form of micronuclei. Although their origin is distinct, DNA damage-induced cytosolic DNA shares common downstream effects and cellular responses with those observed upon viral or bacterial infections. In mammalian cells, multiple factors and enzymes have the capability to sense cytosolic DNA to activate appropriate immune responses. The induction of innate immune responses by the presence of cytosolic DNA not only is crucial for the host organism's first-lines of defense against invading pathogens but also fulfils key oncosuppressive functions through elimination of damaged cells (Vanpouille-Box et al. 2018). Intensive research in recent years has led to the discovery and characterization of the cGAS/STING system as a major component of cells’ intrinsic immune response to the occurrence of cytosolic oligonucleotides. cGAS is a cytosolic nucleotidyltransferase that, upon binding DNA, catalyzes the synthesis of cyclic GMP-AMP (cGAMP) from ATP and GTP, which subsequently acts as a messenger molecule for the adaptor protein STING (Ablasser et al. 2013; Sun et al. 2013; Wu et al. 2013b). cGAS binds double-stranded DNA (dsDNA) in a sequence-nonspecific manner through interaction with the sugar-phosphate backbone, although a preference for specific DNA length (>45 bp) has been observed (Civril et al. 2013; Li et al. 2013; Herzner et al. 2015; Zhou et al. 2018). Upon cGAMP binding, STING, originally located in the endoplasmic reticulum (ER) membrane, translocates to the Golgi apparatus via the ER–Golgi intermediate compartment, while activating at least three distinct kinases—TBK1, IKK, and NIK (TANK-binding kinase 1, IκB kinase, and NFκB-inducing kinase, respectively)—during this process. The activation of these kinases and concomitant phosphorylation of interferon regulatory factor 3 (IRF3) and IκBα transduces the initial stimulus of cytosolic DNA into IRF3 and NFκB transcriptional programs, which control the balance between cellular survival and controlled cell death (Fitzgerald et al. 2003; Ishikawa and Barber 2008; Ishikawa et al. 2009; Tanaka and Chen 2012).

In order to prevent the accumulation of cytosolic DNA and potentially persistent proinflammatory signaling, several enzymes and factors constantly survey and eliminate nucleic acids appearing in the cytosol. One of these, TREX1, is a 3′–5′ exonuclease that acts on either single-stranded DNA (ssDNA) or dsDNA and comprises the predominant enzyme that degrades cytosolic DNA in mammalian cells (Yang et al. 2007; Stetson et al. 2008; Lindahl et al. 2009). Mutations in *TREX1* result in dysregulation of type I IFN production and are associated with the autoimmune disorders Aicardi-Goutières syndrome (AGS) and familial chilblain lupus (Crow et al. 2006a; Rice et al. 2007). Type I IFN responses observed in *TREX1*-deficient cells are dependent on the cGAS/STING system, which presumably recognizes elevated cytosolic DNA arising in the absence of its TREX1-mediated clearance. In line with these observations, inactivation of cGAS/STING abrogates type I IFN signaling in TREX1-deficient cells and rescues the embryonic lethality of *Trex1^−/−^* mice (Stetson et al. 2008; Ablasser et al. 2014; Gray et al. 2015). Notably, individuals with mutations in the *RNase H2* complex, consisting of RNASEH2A, RNASEH2B, and RNASEH2C, show clinical pathologies that resemble those observed in *TREX1* mutated patients, including aberrant type I IFN signaling along with AGS and systemic lupus erythematosus. Although this maladaptive immune response upon impaired RNase H2 function relies on cGAS/STING, as is the case for TREX1 dysfunction, it is unlikely that RNase H2 directly eliminates cytosolic DNA (Crow et al. 2006b; Mackenzie et al. 2016). Rather, RNase H2 functions inside the nucleus by resolving DNA–RNA hybrids and removing ribonucleotides during DNA replication, thereby preserving genome integrity by restricting cytosolic accumulation of broken DNA molecules (Reijns et al. 2011, 2012). Notably, DNA–RNA hybrids harbor the potential to directly stimulate cGAS and, consequently, cGAMP production in vitro (Mankan et al. 2014). Although the induction of cGAMP synthesis in the presence of DNA–RNA hybrids is orders of magnitude lower compared with cGAMP synthesis upon dsDNA stimulation, it may be that DNA–RNA hybrids represent direct stimuli for IFN responses upon RNase H2 dysfunction in certain settings. Recently, it became apparent that the simple model of cGAS being exclusively localized in the cytoplasm might not represent the full picture. Several reports indicated nuclear functions for cGAS, suggesting that a subfraction of the cellular cGAS pool resides within the nucleus (Liu et al. 2018a; Jiang et al. 2019). The concomitant conflict of autoreactivity against self-DNA is reported to be overcome by the tethering of cGAS to the acid patch of nucleosomes via its DNA binding domains, thus blocking DNA binding and activity toward chromatinized DNA (Boyer et al. 2020; Kujirai et al. 2020; Michalski et al. 2020; Pathare et al. 2020; Zhao et al. 2020). This mechanism may also give a plausible explanation to the long-standing question of how cGAS activity is suppressed in mitosis, when the nuclear envelope breaks down and chromosomes are exposed to cytosolic cGAS (Zierhut et al. 2019).

## Impacting cellular immune responses through DDR inhibitors

**Figure 2.**
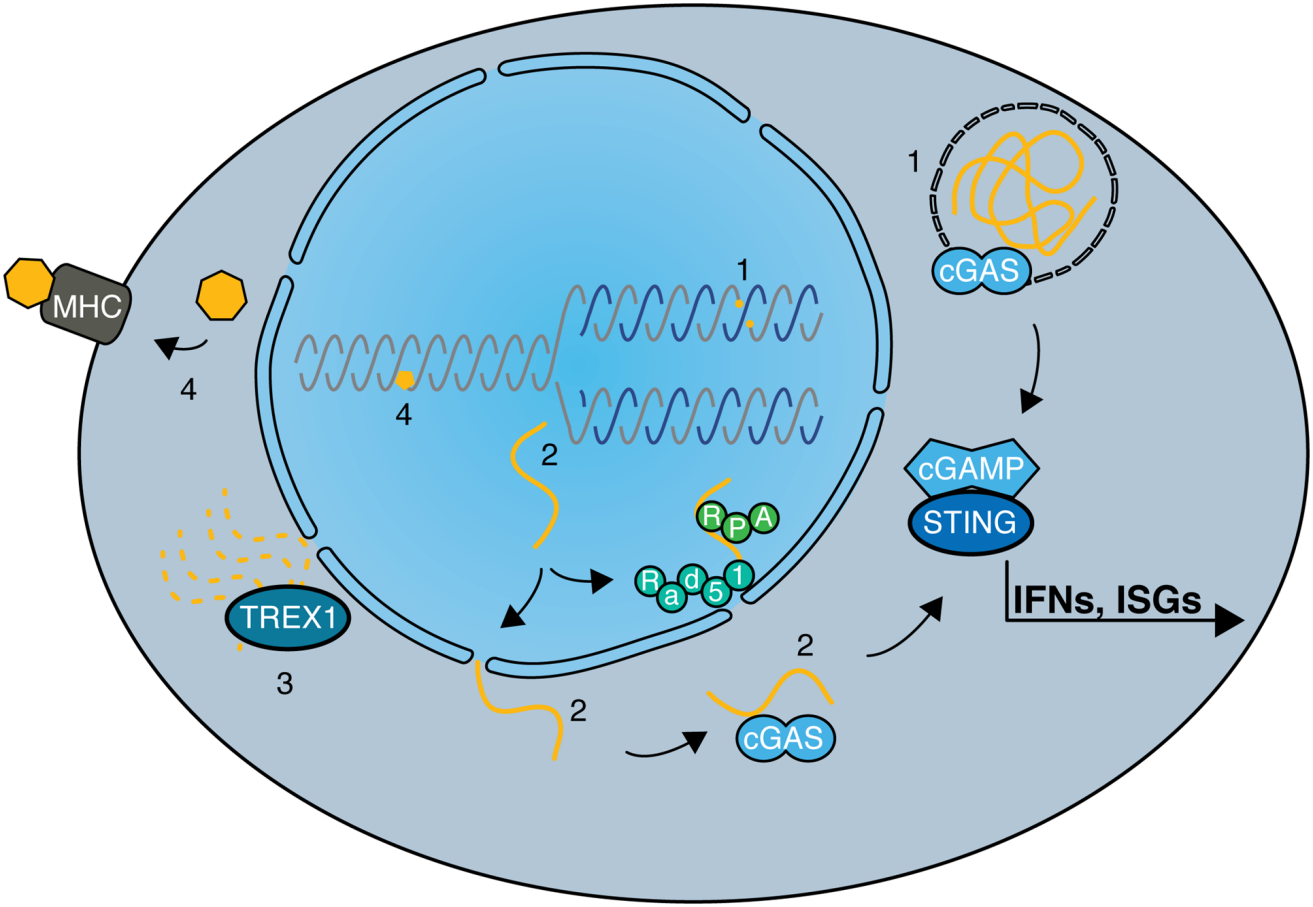
DNA repair defects and their impacts on cellular immune responses. Defects in DNA repair pathways or DDR components affect innate and adaptive immune responses in various ways. (1) The induction of DSBs via chemotherapeutics or irradiation can lead to micronuclei formation and consequent recognition of cytosolic DNA by cGAS/STING. Deficiencies in genes encoding proteins such as BRCA2, RNase H2, or ATM further augment these effects. (2) Cytosolic DNA can also be a result of aberrant processing of DNA replication intermediates, with several DDR factors limiting either the generation (SAMHD1) or the translocation (RAD51 and RPA) of cytosolic DNA from the nucleus, or degrading DNA once it is present in the cytosol (TREX1). (3) Activation of the cGAS-cGAMP-STING cascade leads to IRF3 and NFκB transcriptional programs, resulting in expression of IFN and ISGs, therefore inducing strong innate immune responses. (4) In contrast, MMR defects can lead to adaptive immune responses through increased somatic mutations and consequent synthesis of neoantigens. When presented by MHC molecules at the cell surface, neoantigens elicit a strong T-cell response, dependent on the immunogenicity of the neoantigen.

The importance of cytosolic DNA-stimulated immune responses in the context of cancer therapy is highlighted by the contribution of cGAS/STING to antitumor immunity in response to radiotherapy. Longstanding reports have indicated that, besides generating cytotoxic DNA damage, radiotherapy also induces a tumor-specific immune response that contributes to the efficacy of this therapeutic modality (McBride et al. 2004). Secretion of inflammatory cytokines such as TNF-α, IL-1α, and IL-6 has been observed in response to ionizing radiation, which ultimately leads to adaptive immune responses via CD8^+^ T cells (Hallahan et al. 1989; McBride et al. 2004; Lee et al. 2009; Di Maggio et al. 2015). Recent reports have established that cGAS/STING plays a key role in linking innate and adaptive immune responses after irradiation. Specifically, cGAS/STING mediates increased IFN-β production in the tumor environment to promote antitumor responses upon irradiation. Impaired type I IFN signaling resulting from cGAS/STING inactivation can be bypassed by exogenous supplementation of cGAMP to induce the antitumor efficacy of radiotherapy, further supporting the idea that in response to radiotherapy, cGAS/STING is an initial sensor and signal transducer of antitumor immune responses (Burnette et al. 2011; Woo et al. 2014; Wu et al. 2014; Deng et al. 2014b). From the above, one can assume that the stimulus that activates cGAS/STING after irradiation must originate from cytosolic DNA. Indeed, micronuclei, resulting from irradiation and ensuing incomplete DNA repair, are recognized by cGAS/STING to activate type I IFN signaling (Fig. 2). Importantly, micronuclei formation requires progression through mitosis following DNA-damage induction, wherein the dissolution and ensuing reformation of the nuclear membrane end up enclosing DNA fragments in a micronuclear envelope (Harding et al. 2017; Mackenzie et al. 2017). Although this envelope shares similarities with the normal nuclear envelope, its assembly remains rudimentary, reflecting its fragility that frequently results in the breakdown of envelope integrity and cytosolic exposure of genomic DNA (Liu et al. 2018b). This phenomenon is particularly important in explaining cGAS/STING activation in response to micronucleation, since cGAS recognizes cytosolic DNA rather than the outer structure of a micronucleus (Mackenzie et al. 2017). Interestingly, TREX1 antagonizes immunogenicity of cancer cells following radiation via its own up-regulation and the consequent enhanced degradation of cytosolic DNA. However, continuous low-dose irradiation circumvents TREX1 engagement and promotes cGAS/STING-dependent IFN-β secretion for antitumor immunity (Vanpouille-Box et al. 2017). In addition, TREX1 inhibits the transfer of dsDNA from irradiated tumor cells to dendritic cells (DCs) via tumor-derived exosomes, resulting in dampened cGAS/STING activation and IFN-β production in DCs (Diamond et al. 2018).

Besides radiation-induced antitumor responses, conventional chemotherapy has the potential to stimulate the innate and adaptive immune systems. As described above, HR deficiency (HRD) is a predictor of PARP inhibitor efficacy and PARP inhibitors are particularly cytotoxic in tumors displaying mutations in *BRCA1/2*. In this regard, it is worthwhile mentioning that beside the generation of DNA damage, induction of a STING-dependent antitumor immunity is a considerable feature of PARP inhibitor cancer therapy. Specifically, PARPi-mediated trapping of PARP on DNA lesions appears to be influential for the innate immune response, as the extent of PARP trapping correlates with the magnitude of innate immune signaling (Kim et al. 2020). PARP inhibitor-mediated immunogenicity depends on the activation of cGAS/STING to elicit a cytotoxic T-cell response. Intriguingly, this immune response is augmented upon *BRCA* deficiency, since *Brca1*-proficient mouse tumors show mitigated immunogenicity in response to PARP inhibitors as compared with *Brca1* mutated ones (Ding et al. 2018; Pantelidou et al. 2019). In line with these observations, PARP inhibition led to a strong induction of interferon-stimulated genes (ISGs) in BRCA2-deficient cells compared with their BRCA2-proficient counterpart (Reislander et al. 2019). However, it has been reported that PARP inhibitors have the potential to initiate cGAS/STING-dependent type I IFN production in human cancer cells independent of their *BRCA* gene status (Shen et al. 2019), presumably due to the mitotic defects or increased replication stress caused by PARPi affecting replication fork speed (Maya-Mendoza et al. 2018; Slade 2019). Future studies are required to illuminate the underlying determinants for the differential responses in *BRCA*-proficient and *BRCA*-deficient contexts.

Other chemotherapeutic agents that generate DNA damage also affect cellular immune responses in various ways, often depending on the DNA damage-induced release of cytosolic DNA. For example, the topoisomerase II inhibitor teniposide induces STING-dependent type I IFN signaling and NF-κB activation in a mouse tumor model, with the consequent DC and T-cell activation promoting antitumor responses, including increased immune cell infiltration (Wang et al. 2019). Furthermore, the TOP2 inhibitors doxorubicin and daunorubicin induce IFN-β production in human cancer cell lines via a mechanism shown to be dependent on STING function (Luthra et al. 2017). Notably, doxorubicin treatment enhances CD8^+^ T-cell amplification and infiltration, as well as IFN-γ production, in tumor environments in mice (Mattarollo et al. 2011). Similar to TOP2 inhibitors, topoisomerase I inhibitors trigger potential immunogenicity, exemplified by DC and CD8^+^ T-cell activation in mice treated with topotecan (Kitai et al. 2017).

Despite lacking clear evidence for affecting the cellular immune system, ATR inhibitors have been shown to potentiate immune stimulations in response to radiotherapy. Thus, combinatorial treatment of radiotherapy and ATR inhibition was found to induce type I/II IFN-based gene expression changes and CD8^+^ T-cell infiltration in a manner dependent on cGAS/STING (Vendetti et al. 2018; Dillon et al. 2019; Sheng et al. 2020). While ATR inhibitors do not damage DNA directly, it can be assumed that the increased immunogenicity in irradiated tumors in the context of ATRi results from overriding the G2/M cell cycle checkpoint, with an increased proportion of cells with unrepaired DNA lesions entering mitosis, leading to DNA fragmentation and micronuclei formation capable of triggering innate immune responses (Ruiz et al. 2016; Harding et al. 2017). In line with this model, it has been observed that inhibition of the ATR effector kinase CHK1 abrogates the G2/M checkpoint post irradiation, leading to micronuclei formation and type I IFN signaling in cancer cells (Chao et al. 2020). Moreover, increased CD8^+^ T-cell infiltration and tumor volume reduction was observed in mice treated with a combination of radiotherapy and the CHK1 inhibitor AZD7762 compared with treatments with these agents individually (Chao et al. 2020). Similar to ATR inhibitors, pharmacologic inhibition of ATM in combination with radiotherapy was found to induce type I IFN signaling, which notably occurred independent of cGAS/STING but was reliant on TBK1 (Zhang et al. 2019).

## Consequences of DDR defects for immune responses

The involvement of DDR factors and their inhibition in the induction of innate immune responses has been highlighted by the consequences of RNase H2 dysfunction in the autoimmune disorder AGS. While certain DDR factors such as TREX1 participate in the sensing of extranuclear DNA, others influence cellular immunity via more indirect mechanisms. The tumor suppressor BRCA2 has a pivotal role in HR-mediated repair and in the protection of stalled replication forks, while its absence is accompanied by genome instability and chromosome breakage (Prakash et al. 2015). Because BRCA2 loss is highly detrimental to cell viability, BRCA2-deficient cancer cells have invariably undergone adaptation processes in order to survive, exemplified by the inactivation of p53 functions. Importantly, alongside rewiring of DNA repair processes, the absence of BRCA2 causes enhanced phosphorylation and therefore activation of STAT1-IRF3, followed by concomitant up-regulation of ISGs. Mechanistically, this adaptive immune response is dependent on cGAS/STING, with BRCA2-deficient cells exhibiting an increase in cGAS-positive micronuclei compared with BRCA2-proficient cells, presumably as result of the DNA repair defects and chromosome instability observed in the absence of BRCA2 function (Reislander et al. 2019). In line with these observations, elevated secretion of proinflammatory cytokines, such as TNF-α, arises as a consequence of BRCA2 loss, resulting from cytosolic DNA sensing by cGAS/STING and ensuing interferon responses (Heijink et al. 2019). Additionally, it has been shown that in the absence of BRCA2, RNase H2 recruitment to DSBs is impaired, resulting in increased DNA–RNA hybrid formation and thus providing and additional explanation for cGAS/STING activation in BRCA2-deficient cells (D'Alessandro et al. 2018). Importantly, the abrogation of TNF-α signaling improves viability of BRCA2-deficient cells, indicative of TNF-α signaling promoting cell death when BRCA2 function is impaired (Heijink et al. 2019). Besides BRCA2 deficiency causing induction of interferon responses and an increase in TNF-α signaling per se, it also further sensitizes cells to autocrine TNF-α signaling.

Notably, depletion of BRCA1 or the inter-strand DNA cross-link (ICL) repair factor FANCD2 sensitizes cells to recombinant TNF-α, suggesting that the general impairment of HR repair or replication stress arising from compromised ICL repair harbors the potential to make cells more susceptible to interferon responses (Heijink et al. 2019). The idea of unprotected replication forks comprising an entry point for aberrant DNA processing and consequent leakage into the cytosol is further supported by the report that mutations in *SAMHD1* are causative for the autoimmune disorder AGS (Crow and Manel 2015). Besides its role as a dNTPase (deoxynucleotide triphosphohydrolase), SAMHD1 promotes the controlled degradation of newly synthesized DNA at stalled replication forks via the exonuclease MRE11. In the absence of SAMHD1, nascent DNA at stalled replication forks is displaced by the RECQ1 helicase and translocates to the cytosol, where it activates the cGAS/STING pathway and ensuing type I IFN responses (Coquel et al. 2018).

In order to avoid chronic proinflammatory signaling as occurs in AGS patients, several cellular mechanisms have evolved to counteract or minimize leakage of DNA fragments into the cytosol. The DNA repair proteins RPA and RAD51 both have the capability to directly bind ssDNA, which is crucial for their functions during DNA replication and DNA repair (Bhat and Cortez 2018). In addition, they prevent the accumulation of cytosolic DNA by binding and therefore retaining ssDNA within the nucleus, thereby working in cooperation with nuclear membrane-bound TREX1, which normally swiftly degrades any DNA leaking into the cytosol (Fig. 2). Exhaustion of the available RPA/RAD51 pool in the absence of TREX1, or upon depletion of RPA or RAD51, results in cGAS/STING-dependent type I IFN signaling (Wolf et al. 2016). Additionally, RAD51 protects newly synthesized DNA from aberrant processing by MRE11 and consequent cytosolic DNA translocation and ensuing cGAS/STING activation (Bhattacharya et al. 2017). Notably, the functions of MRE11 as a nuclease that processes DNA replication fork intermediates can be seen as a double-edged sword. On one hand, MRE11 prevents cGAS/STING activation through trimming of replication intermediates, stimulated by SAMHD1. On the other hand, excessive processing of unprotected, stalled replication forks in the absence of RAD51 by MRE11 generates cytosolic DNA and consequently stimulates cGAS/STING. Whether or in which form RAD51 cooperates with SAMDH1 for fine-tuning of this process is an interesting area for future research. Besides MRE11-dependent degradation of nascent DNA at stalled or collapsed replication forks, other DNA nucleases can process DNA structures in such contexts, subsequently leading to activation of innate immune responses via cytosolic DNA sensing. In particular, the actions of the structure-specific endonuclease MUS81 can bring about the accumulation of cytosolic DNA via MUS81-dependent cleavage of stalled replication forks, leading to cGAS/STING activation and induction of type I IFNs, as observed in various prostate cancer cell lines (Ho et al. 2016). Moreover, the MUS81–STING axis is responsible for a prostate cancer cell-specific T-cell response in mice, thus highlighting a likely role for MUS81 in promoting antitumor immunity. In addition, endogenously arising DSBs are capable of stimulating innate immune responses in situations where accurate DNA repair is compromised. Accordingly, deficiency in ATM induces type I IFNs in unchallenged conditions, indicative of spontaneously arising DNA DSBs being the stimulus of this immune response. Furthermore, in the absence of ATM, ssDNA resulting from unrepaired DNA lesions accumulates in the cytoplasm and activates cGAS/STING-dependent type I IFN signaling (Härtlova et al. 2015). Notably, this DDR defect primes type I IFNs to enhance the innate immune response toward invading pathogens, highlighting the fact that damaged DNA serves as a danger signal for cellular homeostasis and prepares the innate immune system for a rapid response in the face of bacterial challenges (Härtlova et al. 2015).

## Mismatch repair deficiency and adaptive immunity

**Figure 3.**
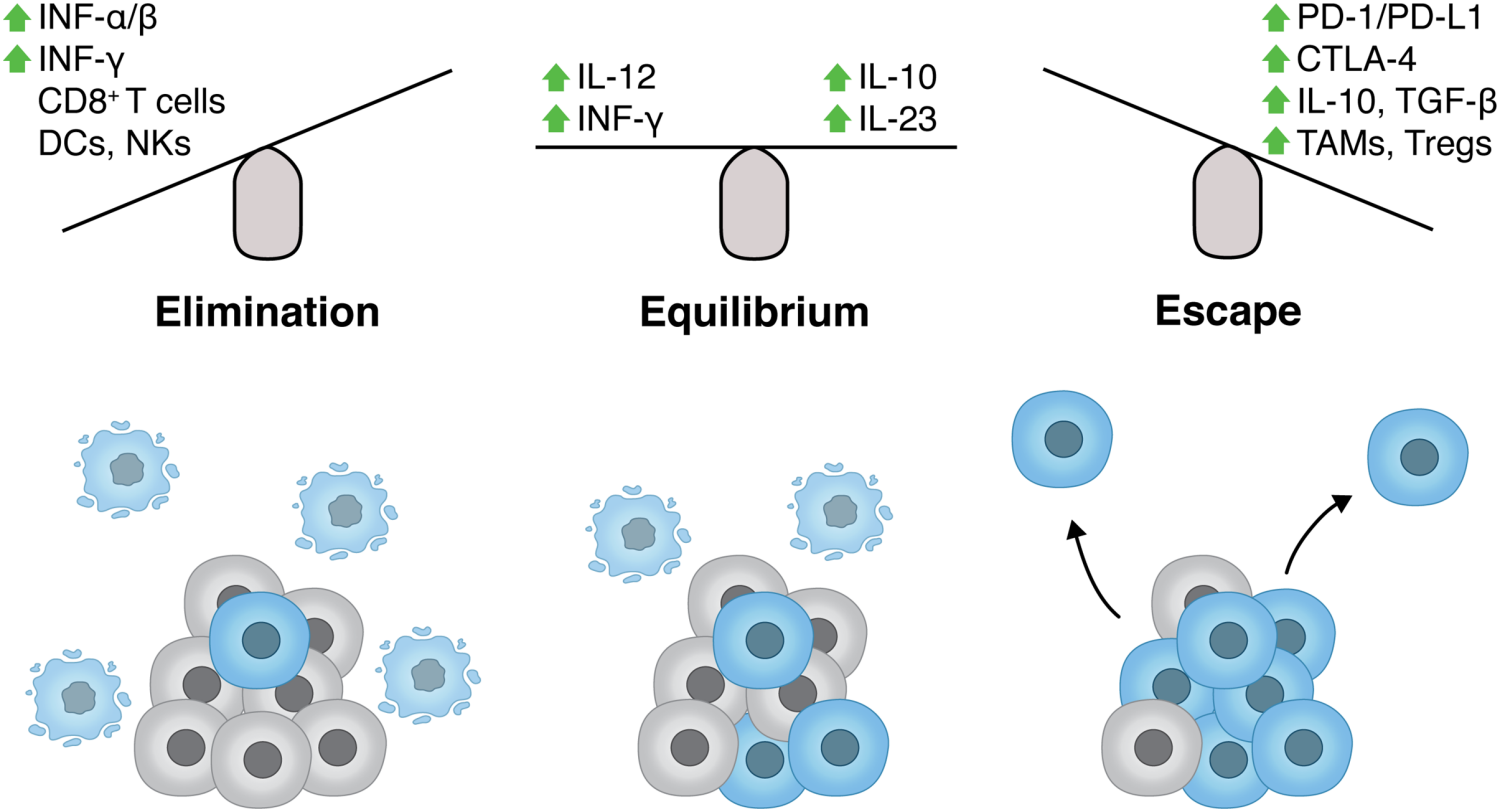
Innate and adaptive immune responses in cancer. In recent years, the concept of cancer immunoediting evolved from traditional views of the immune system constantly surveying and eliminating transformed cells in order to counteract cancer development (immunosurveillance) (Keast 1970). Cancer immunoediting unifies observations of the immune system promoting tumor outgrowth with reports of immunosurveillance, highlighting the dual functions of the immune system during tumor development (Schreiber et al. 2011). The cancer immunoediting concept consists of three phases: elimination, equilibrium, and escape. In the elimination phase, components of both the innate and adaptive immune response recognize and destroy cells undergoing oncogenesis. Elimination is promoted by a number of signaling molecules such as type I and type II IFNs and is executed via the interplay of a subset of immune cells such as CD8^+^ T cells, dendritic cells (DCs), natural killer cells (NKs), natural killer T cells (NKTs), proinflammatory (“M1”) macrophages, and others (Mittal et al. 2014). Notably, the DDR participates in this process, since DNA damage induction in tumors cells results in up-regulation of ligands for the receptors NKG2D and DNAM-1, therefore stimulating cytotoxicity of NK and CD8^+^ T cells in addition to IFN-γ secretion (Gasser et al. 2005; Croxford et al. 2013). Moreover, radiotherapy-induced DNA damage, and consequent cell death due to uncomplete DNA repair, stimulates cross-presentation by dendritic cells and increased lymphocyte influx, thus further contributing to cancer cell elimination (Deng et al. 2014b; Samstein and Riaz 2018; Cornel et al. 2020; Cheng et al. 2021). Paradoxically, TNF-α has both antitumor and tumor-promoting activity. When secreted by macrophages and innate immune cells, TNF-α induces cancer cell elimination, whereas chronic inflammation promoted by TNF-α signaling can drive carcinogenesis (Balkwill 2009; Charles et al. 2009). In the equilibrium phase, the adaptive immune system holds the tumor in a dormant state with cancer cells resisting constant immune recognition through genetic and epigenetic changes in antigen presentation and immunosuppressive pathways. Cancer cells achieve immune evasion by various mechanisms, including loss of tumor antigens or factors involved in antigen presentation, such as type I HLA (MHC) function, expression of inhibitory ligands (e.g., PD-L1 and CTLA-4), secretion of immunosuppressive cytokines (IL-10, TGF-β), and recruitment of tumor-associated macrophages (TAMs) and regulatory T cells (Tregs). These scenarios result in the inability of innate and adaptive immune cells to recognize and appropriately respond to oncogenic cells, therefore facilitating tumor progression (escape phase) (Vinay et al. 2015).

Deficiencies in DNA repair pathways can also affect adaptive immune responses, which are key for immunogenicity of tumors and have clinical implications in the context of cancer immunotherapy. Additionally, DNA damage responses are crucial during the development of the immune system and maturation of immune cells, as exemplified by the controlled induction and concomitant repair of DSBs during V(D)J recombination and class switch recombination in lymphocytes (for an extensive review, see Bednarski and Sleckman 2019). Furthermore, over recent years, a connection between defects in the DNA mismatch repair (MMR) pathway and tumor immunogenicity has been observed both in preclinical model systems and in cancer patients. The MMR machinery detects and replaces base mismatches resulting from erroneous DNA replication or repair and, in particular, plays key roles in correcting small insertion or deletions (indels) arising at repetitive sequences in the genome, so-called microsatellite instability (Kunkel and Erie 2015). Tumors harboring mutations in genes encoding the core MMR factors MLH1, MSH2, MSH6, or PMS2 are characterized by microsatellite instability (MSI), a hypermutator phenotype specified by large numbers of single-nucleotide variants (SNVs) and indels, thus leading to a high mutational burden. Additionally, specific DNA proofreading mutations in the replicative DNA polymerases POLE and POLD1 drive hypermutation phenotypes in some cancers without causing MSI (Campbell et al. 2017). MSI predominantly occurs in endometrial, gastric, and colorectal cancers, suggesting that such tissue types or the tumor environment and extracellular influences affect MSI development (Hause et al. 2016; Cortes-Ciriano et al. 2017). The correlation between MSI colorectal tumors and high numbers of tumor-infiltrating lymphocytes has long been acknowledged. Colorectal MSI tumors frequently display high infiltration of CD8^+^ cytotoxic T cells, type 1 helper (Th1) cells, and memory T cells, alongside the up-regulated expression of interferon γ (IFN-γ) and immune checkpoint molecules PD-1, PD-L1, and CTLA4 (Fig. 3; Llosa et al. 2015; Mlecnik et al. 2016).

**Figure 4.**
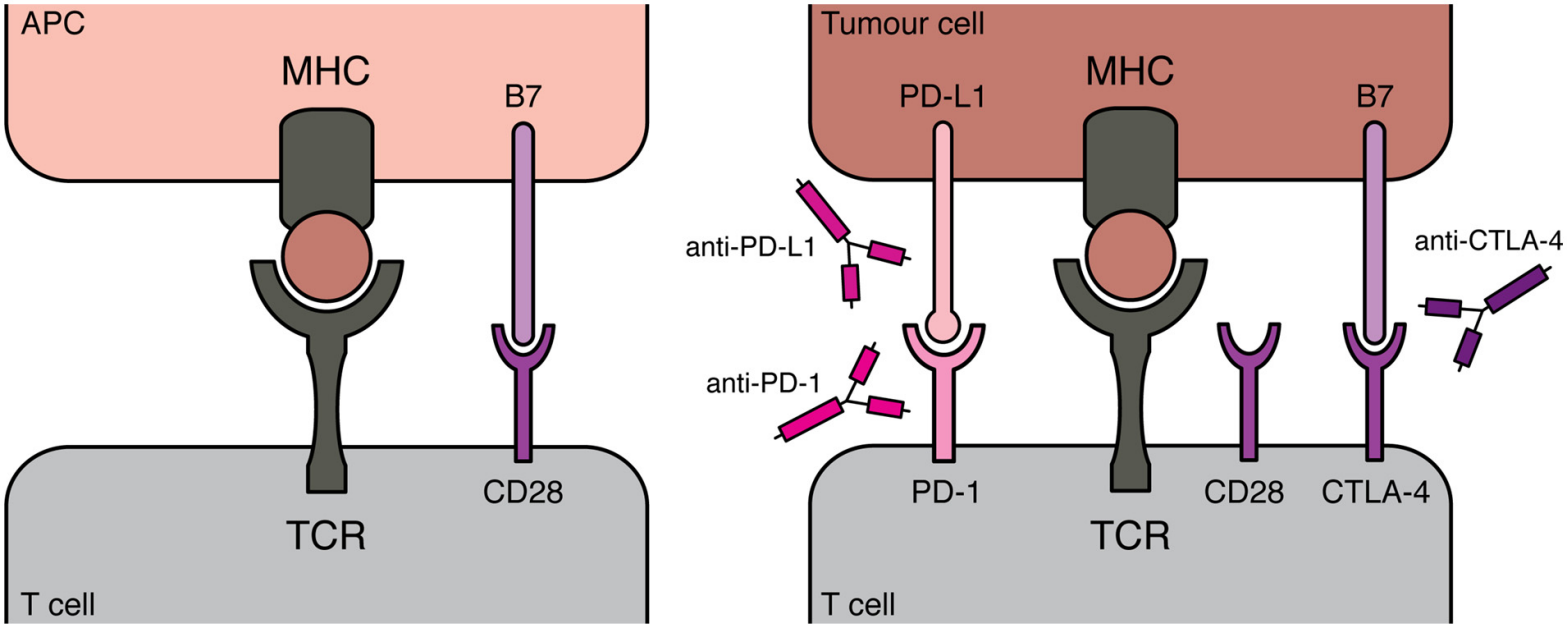
Principles of immune checkpoint inhibitors for cancer therapy. As described in Figure 3, during the escape phase of the immunoediting concept, tumor cells evade immune recognition and destruction by active immunosuppression in the tumor. A milestone for the field of immune checkpoint inhibitors was the report in which melanoma patients treated with an antibody targeting the T-cell checkpoint protein CTLA-4 showed significantly improved survival compared with the control group (Hodi et al. 2010). This suggested that targeting suppressive immune checkpoints can improve overall survival in melanomas, indicating that a patient's immune system has capabilities to control tumor growth once immunosuppressive signals are overcome. Since this pharmacological approach targets the patient's immune system rather than the tumor itself, a new field for clinical research arose. CTLA-4 is a surface receptor on T cells. To acquire effector function, a T-cell recognizes its compatible antigen, presented by MHC molecules of an antigen-presenting cell (APC), via its T-cell receptor (TCR). However, this initial recognition is insufficient, with binding of the CD28 T cell receptor to B7 molecules (CD80 or CD86 ligand) on APCs serving as crucial costimulatory signals to adequately prime T cells. CTLA-4 translocates to the cell surface once T cells are activated, where it binds CD80 and CD86 with higher affinity than CD28, therefore dampening T-cell activation (Walunas et al. 1994; Krummel and Allison 1995). Moreover CTLA-4 expression by Tregs is crucial for their immune suppressive functions, potently binding CD80/CD86 ligands on APCs and therefore preventing T-cell activation (Takahashi et al. 2000; Wing et al. 2008). Following its initial success in clinical trials, the CTLA-4 antibody ipilimumab was FDA approved in 2011 for treating melanomas. PD-1 represents another inhibitory receptor present on T cells, while its ligands PD-L1 and PD-L2 can be expressed by various cell types, including APCs and malignant cells, predominantly after exposure to inflammatory cytokines such as IFN-γ. Engagement of PD-L1 with its receptor PD-1 interferes with TCR signaling, therefore limiting T-cell responses toward tumor cells (Freeman et al. 2000; Dong et al. 2002). Following this rational, antibodies targeting PD-1/PD-L1 have provoked clinical benefits in various types of cancers, warranting FDA approval of pembroluzimab and nivolumab (both PD-1 antagonists) in 2014. Unlike CTLA-4, PD-1/PD-L1 does not interfere with costimulation during the T-cell activation, suggesting that combination therapy of CTLA-4 and PD-1/PD-L1 antibodies could have synergistic therapeutic effects. Regaining T-cell activation, by blocking inhibitory signals during costimulation via CTLA-4 antibodies, could drive increased PD-L1 expression in tumor cells, making them particularly susceptible to PD-1/PD-L1 checkpoint blockade (Sharma and Allison 2015).

MSI endometrial tumors also exhibit increased numbers of tumor-infiltrating lymphocytes and elevated expression of PD-1/PD-L1 compared with microsatellite stable (MSS) tumors (Howitt et al. 2015). Recently, this connection has been attributed to the increase in neoantigens due to the high mutational burden in these settings (Tougeron et al. 2009; Maby et al. 2015). To a certain degree, it seems reasonable to assume that the more mutations in the genome, the more likely it is that neoantigens are formed and in due course recognized by the immune system (Fig. 2; Turajlic et al. 2017). In line with this idea, increased mutational burden correlates with improved survival in colorectal cancer patients (Giannakis et al. 2016). Interestingly, additional tumor types such as melanomas and lung cancers, despite being microsatellite stable (MSS), exhibit an increased number of overall mutations compared with others, most likely due to exposure to exogenous mutagens, including ultraviolet light and tobacco smoke components, respectively, suggesting that these may also promote antitumor immune responses (Alexandrov et al. 2013). In accord with this idea, a recent study by Bardelli and colleagues (Germano et al. 2017) demonstrated that colorectal, breast, and pancreatic mouse cancer cell lines, where MMR was genetically inactivated, grew significantly slower when transplanted into immunocompetent mice compared with the isogenic MMR-proficient cancer cell lines, indicating rejection by the host immune system. Enhanced immunosurveillance was accompanied by accumulation of neoantigens over time in MMR-deficient cells, wherein the amount of neoantigens remained stable in MMR-proficient cells, implicating neoantigens generated by MMR deficiency as a direct cause for immune system-mediated elimination of cancer cells (Germano et al. 2017). Importantly, fully established MMR-deficient tumors are often sensitive to immune checkpoint inhibitors in people, which points toward MMR deficiency as an important determinant of immune checkpoint blockade (Fig. 4) efficacy, at least in certain cancer types (Le et al. 2015).

## Combined targeting of DDR and immune checkpoints in cancer therapy

Immune checkpoint inhibition (or immune checkpoint blockade [ICB]) has experienced considerable success in recent years as a promising therapeutic strategy for a subset of cancers, presenting an alternative to irradiation therapy or classical chemotherapies (Fig. 4; Ribas and Wolchok 2018). In particular, their potential to counteract immune suppressive signals in the tumor microenvironment to overcome T-cell exhaustion has been proven to be beneficial in clinical settings (Fig. 3). ICBs target immune checkpoints, which in normal settings are important to accurately regulate T-cell activation and T-cell receptor signaling, thus preventing chronic (or inappropriate) immune responses. In cancers, these checkpoints, often engaged via cell surface ligands or receptors, are repurposed to dampen antitumor immune responses and create an immune-suppressive tumor microenvironment. ICB agents, in the form of antibodies, binding to these ligands/receptors (anti-CTLA-4 and anti-PD-1/anti-PD-L1) can overcome inhibitory signaling and reactivate T-cell engagement toward the tumor (Fig. 4). In order to broaden the spectrum of applications for ICB, extensive efforts have centered around the identification of suitable biomarkers for predicting ICB efficacy, as well as exploring the potential of combination therapies with conventional irradiation or chemotherapy. Increased mutational load is a promising indicator of ICB responsiveness, and indeed, patients harboring mutations in MMR genes and displaying MSI showed strong responsiveness to PD-1 antagonists (Rizvi et al. 2015; Le et al. 2017; Overman et al. 2017). Importantly, the clinical benefits of blocking the PD-1/PD-L1 immune checkpoint occurred across various tumor types, including melanomas, colorectal, and non-small cell lung cancers (NSCLCs), highlighting the likelihood that increased mutational burden was the common denominator of ICB efficacy between these tumors. In line with this premise, progression free survival and overall survival upon PD-1 blockade was further augmented when combined with a CTLA-4 antagonist in small cell lung cancers, which exhibit a high load of somatic mutations (Hellmann et al. 2018).

## Synergy of PARP inhibitors with immune checkpoint inhibition

In addition to being used to treat tumors with a high mutational burden, ICBs have also been shown to be effective when applied in combination with DDR inhibition (Table 2). An important feature of PARP inhibition is that it is associated with increases in CD8^+^ T-cell infiltration and IFN-γ production in the tumor, which occurs in mouse cancer models independent of their *Brca1/2* status. Moreover, tumor regression was further increased upon combination therapy of PARPi with anti-PD-1 antibodies compared with the respective monotherapies (Ding et al. 2018; Shen et al. 2019). Initial results of ensuing clinical trials indicate beneficial effects in a subset of patients treated with ICB and PARPi, with results from the MEDIOLA trial suggesting increased antitumor activity in germline *BRCA* mutated ovarian cancers treated with the combination therapy of olaparib and durvalumab (PD-L1 inhibitor) (Domchek et al. 2020).

**Table 2.**
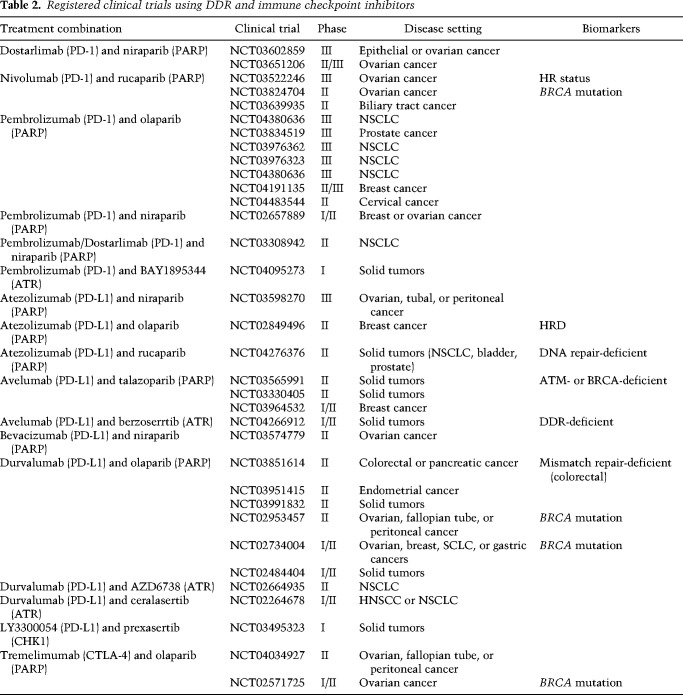
Registered clinical trials using DDR and immune checkpoint inhibitors

Another clinical trial, TOPACIO, investigated the efficacy of the PARPi niraparib and the PD-L1 inhibitor pembrolizumab in platinum-resistant advanced breast cancers and recurrent ovarian cancers, independent of their HR proficiency. Notably, patient response toward the combination therapy in ovarian cancers exceeded expectations based on monotherapy efficacy, while HRD-associated mutational signature 3 or a positive immune score were reliable indicators of responsiveness (Konstantinopoulos et al. 2019; Färkkilä et al. 2020). Although mutational signature 3 and therefore HR proficiency predicted efficacy of this combination therapy, therefore implying an application for niraparib and pembrolizumab in BRCA-deficient tumors, it was also encouraging that PD-L1 presence and interferon priming of immune cells present in the tumor microenvironment can be used to estimate responsiveness to the combination therapy independent of HRD. Indeed, patient responses to PARPi and PD-L1 inhibition have been observed in tumors displaying a functional HR pathway (Färkkilä et al. 2020). Additionally, a proof-of-concept clinical study further confirmed immune activation in ovarian cancers treated with olaparib and the anti-PD-L1 antibody durvalumab regardless of their *BRCA* status, highlighted by increased IFN-γ production and tumor-infiltrating lymphocytes (Lampert et al. 2020). Notably, in this study no significant increase in tumor mutational burden was observed (not even in *BRCA*-deficient tumors), which is in line with previous observations in ovarian cancers and suggests that immune stimulation with concomitant PARPi treatment is unlikely to result from the exposure of neoantigens (Chan et al. 2019). However, increased proinflammatory cytokine expression and T-cell activation was associated with clinical benefits following olaparib plus durvalumab treatment, implying a potential application of this therapy for a wider spectrum of cancers. Currently, numerous clinical trials are evaluating the efficacy of PARP inhibitors in combination with ICB (Table 2). Although a significant proportion of these studies are preselecting patients based on potential DDR defects, it will be valuable to compare outcomes with trials where patients received the combination therapy independent of DDR deficiencies. Clarifying whether and to what extent DDR deficiency promotes responses to PARPi + ICB, and whether mutations in DDR genes (or mutational signatures characteristic for DDR deficiencies) can serve as reliant biomarkers for predicting PARPi + ICB efficacy will be crucial for the clinical prospects of this combination therapy approach.

## Potentiating immune checkpoint inhibitor efficacy via DDR inhibitors

Beyond PARP inhibitors, combinations of DNA-damage inducing agents with ICB have shown encouraging preclinical/clinical results, as exemplified by the recent FDA approval of atezolizumab (anti-PD-L1) in combination with carboplatin and etoposide for extensive-stage small cell lung cancers (SCLCs). Prior to this approval, the Impower133 clinical trial had shown significantly longer overall and progression-free survival in small cell lung cancer (SCLC) patients subjected to this combination therapy compared with chemotherapy alone (Horn et al. 2018). Interestingly, in this study there was no indication of higher mutational burden being beneficial for patient response, opposing preclinical studies suggesting positive correlation between mutational load and ICB monotherapy (Hellmann et al. 2018). Since the Impower133 trial cohort represented patients with advanced disease, it is possible that tumor progression selects against strong immunogenic neoantigens. Consequently, the efficacy of combination therapy could result from chemotherapy-induced DNA damage and induction of inflammatory responses in the tumor microenvironment. Other DDR inhibitors being assessed for synergistic effects with immune modulating agents are still at the stage of preclinical investigations. In this regard, it is notable that inhibiting PARP or CHK1 in the presence of anti-PD-L1 antibodies caused tumor regression in SCLC mouse models, which was accompanied by CD8^+^ T-cell infiltration (Sen et al. 2019b). Further analysis revealed that PARP inhibition caused a drastic increase in PD-L1 and IFN-β expression in tumors, while the antitumor effect of either CHK1i + anti-PD-L1 or PARPi + anti-PD-L1 was dependent on functional cGAS/STING. Additionally, combining the TOP1 inhibitor irinotecan with PD-1/PD-L1 antibody mildly reduced tumor growth in cancer mouse models (Iwai et al. 2018; McKenzie et al. 2018; Sen et al. 2019a).

As we described above, radiation therapy has multiple impacts on immune responses in cancer cells, resulting in cGAS/STING activation via cytosolic DNA, leading to type I IFN responses. Therefore, it appears that ICBs have the potential to augment anti-immune responses upon radiotherapy of tumors. Indeed, complementing radiotherapy with anti-PD-L1 antibody treatment extended the efficacy of radiotherapy in mouse tumor models via cytotoxic T-cell activation, accompanied by reduced infiltration of immune suppressive myeloid cells (Deng et al. 2014a). Furthermore, a phase I clinical trial revealed major tumor regression in a group of metastatic melanoma patients treated with radiotherapy and an anti-CTLA-4 antibody (Twyman-Saint Victor et al. 2015). Importantly, this effect was reproduced in mouse models, although resistance was observed after a certain time, likely caused by up-regulation of PD-L1 expression in melanoma cells, leading to T-cell exhaustion. In line with these observations, patients displaying high levels of PD-L1 did not response to radiotherapy + anti-CTLA-4 therapy, but inhibition of the PD-1/PD-L1 axis in these tumors re-engaged exhausted T cells and improved the efficacy of radiotherapy + anti-CTLA-4 (Twyman-Saint Victor et al. 2015). Moreover, a phase III study in non-small cell lung cancer (NSCLC) patients revealed increased progression-free survival when radiotherapy was combined with the PD-L1 inhibitor durvalumab compared with the control group (Antonia et al. 2017). An important feature of radiotherapy is its potential to induce tumor regression distant from the site of radiation, which depends on the host's immune system; termed the “abscopal effect.” Although this effect occurs only sporadically, there is a consensus that immune checkpoint inhibitors have the potential to systematically promote abscopal effects in tumors (Ngwa et al. 2018). In mouse models, sensing of cytosolic DNA via cGAS/STING was shown to be required for the abscopal effect observed in response to radiotherapy and CTLA-4 inhibitors (Harding et al. 2017). In line with these observations, combining radiotherapy with ipilimumab (anti-CTLA-4) induced systemic antitumor immune responses in NSCLC patients, indicative of abscopal effects. Increased IFN-β secretion and clonal expansion of tumor-derived T-cell receptors (TCRs) predicted responsiveness to this combination therapy (Formenti et al. 2018), thereby highlighting the potential of radiotherapy to induce type I IFN responses promoting innate antitumor immunogenicity. In conclusion, innate immunity is fundamental to creating an adaptive immune response in immunosuppressive tumors, and the induction of DNA damage via DDRis or radiotherapy represents a potent trigger of this response.

## Concluding remarks

It is becoming increasingly clear that DNA damage and activation of DNA damage responses can result in profound stimulation of aspects of the immune system. Defects in DNA repair pathways such as HR as well as DNA replication stress and concomitant aberrant processing of DNA replication forks can trigger cytosolic DNA sensing via cGAS/STING, leading to robust interferon responses. Furthermore, DDR inhibitors harbor the potential to activate antitumor immune responses and contribute to their therapeutic efficacy. The abrogation of cytosolic DNA sensing via cGAS/STING and proinflammatory signaling diminishes PARPi cytotoxicity in mouse tumor models, indicative of uncompleted DNA repair products being the initial stimulus of this immune response. Since comparable immune stimulation from PARPi can be observed in clinical scenarios, particularly when their application is combined with immune checkpoint inhibitors, it will be crucial to define and understand the exact circumstances wherein DNA lesions can activate antitumor immunity. Our current understanding suggests that in some scenarios, DDR inhibition or radiotherapy induces proinflammatory signaling events in tumor-suppressive microenvironments, which, when combined with ICB, promote T-cell-mediated destruction of tumor cells. It is tempting to speculate that the sheer presence of aberrant DNA repair products, rather than mutational burden and resulting neoantigens, is the key stimulus for these immune responses. Importantly, this could imply efficacy of DDR inhibitors and ICB treatment combinations in advanced disease settings, where a strong immune suppression of the tumor milieu frequently suppresses antitumor immunity. Ongoing and future clinical trials will shed light on these issues and may identify biomarkers for predicting a patient's responsiveness to various potential therapies, thereby helping tailor therapies to individual patients and impacting on the future clinical success of combining DDR inhibitors with ICBs. Importantly, in the context of MSI tumors, DDR defects can be a source for mutational burden and the consequent generation of neoantigens in tumor cells. The prevailing clinical success of ICB in MSI or other high mutational burden tumors opens the therapeutic avenue of combining ICB with MMR inhibitors for targeted application in patients independent of the mutational load in the tumor. Finally, the development and clinical exploration of inhibitors of DDR components such as ATR, ATM, and DNA-PK will shed more light on how these inhibitors potentially affect cellular immune responses and how these might be exploited in combinations with immune-targeting therapies. Furthermore, inhibitors targeting DNA repair polymerases, such as POLθ, represent an exciting arena for future studies, not only in the context of DDR alone but also in regards to their potential interplay with the cellular immune systems (Schrempf et al. 2020). In this regard, it is of note that preclinical studies with POLθ inhibitors have indicated promising efficacies in tumor models, reminiscent of the initial studies when PARP inhibitors were being developed (Zhou et al. 2020). Given the widespread efforts in both the DDR inhibitors and ICB arenas and the interest in their synergies, it seems likely that we will soon learn of other contexts where their combination will enhance the health and well-being of cancer patients.
